# miR-27b shapes the presynaptic transcriptome and influences neurotransmission by silencing the polycomb group protein Bmi1

**DOI:** 10.1186/s12864-016-3139-7

**Published:** 2016-10-04

**Authors:** Vivian Y. Poon, Minxia Gu, Fang Ji, Antonius M. VanDongen, Marc Fivaz

**Affiliations:** 1 Program in Neuroscience and Behavioral Disorders, Duke-NUS Medical School, Singapore, Singapore; 2 Department of Physiology, Yong Loo Lin School of Medicine, National University of Singapore, Singapore, Singapore

**Keywords:** miRNAs, Synapse, Transcriptome, Polycomb Group Protein, Neurotransmission, Epigenetics

## Abstract

**Background:**

MicroRNAs (miRNAs) are short non-coding RNAs that are emerging as important post-transcriptional regulators of neuronal and synaptic development. The precise impact of miRNAs on presynaptic function and neurotransmission remains, however, poorly understood.

**Results:**

Here, we identify miR-27b—an abundant neuronal miRNA implicated in neurological disorders—as a global regulator of the presynaptic transcriptome. miR-27b influences the expression of three quarters of genes associated with presynaptic function in cortical neurons. Contrary to expectation, a large majority of these genes are up-regulated by miR-27b. This stimulatory effect is mediated by miR-27b-directed silencing of several transcriptional repressors that cooperate to suppress the presynaptic transcriptome. The strongest repressive activity appears to be mediated by Bmi1, a component of the polycomb repressive complex implicated in self-renewal of neural stem cells. miR-27b knockdown leads to reduced synaptogenesis and to a marked decrease in neural network activity, which is fully restored by RNAi-mediated silencing of Bmi1.

**Conclusions:**

We conclude that silencing of Bmi1 by miR-27b relieves repression of the presynaptic transcriptome and supports neurotransmission in cortical networks. These results expand the repressive activity of Bmi1 to genes involved in synaptic function and identify a unique post-transcriptional circuitry that stimulates expression of synaptic genes and promotes synapse differentiation.

**Electronic supplementary material:**

The online version of this article (doi:10.1186/s12864-016-3139-7) contains supplementary material, which is available to authorized users.

## Background

miRNAs are small noncoding RNAs that regulate gene expression by base pairing to mRNAs. Targeting occurs by complementarity between the seed region (6–8 bp) of the miRNA and the mRNA 3’ UTR [1], and results in transcript silencing by translational repression and mRNA destabilization [2–4]. This minimal degree of base pairing enables a single miRNA to target multiple (tens to hundreds) transcripts [5, 6] thereby complicating the identification of relevant miRNA targets for a given biological outcome. On the other hand, a single gene is typically under the control of several different miRNAs. Consequently, miRNA networks appear highly distributed, with each individual miRNA having a widespread but modest effect on gene expression.

miRNAs are abundant in the brain and have been implicated in various aspects of CNS development, from renewal of neural stem cells, to differentiation, neurite projection, dendrite maturation and synaptic plasticity [7]. The impact of miRNAs on neurodevelopment in both vertebrates and invertebrates is now supported by a large body of work (reviewed in [7]), and provides a plausible explanation for the increasing number of miRNAs associated with neurodevelopmental disorders [8]. Recent studies also point to a role of miRNAs at the synapse, particularly on the post-synaptic side. Specific miRNAs control dendrite complexity [9], dendritic excitability [10, 11] and spine shape [12–14], while others target glutamate receptors [15, 16] and post-synaptic scaffolds [17], or regulate Long-Term Potentiation (LTP) and Depression (LTD) [18, 19], persistent forms of synaptic plasticity that are thought to rely predominantly on post-synaptic mechanisms.

Comparatively less is known about the effect of miRNAs on presynaptic functions and neurotransmitter release. This is somewhat surprising, given that proper dosage of neurotransmitter release is of central importance for neural circuit performance and is fine-tuned by complex gene regulatory networks [20]. In support, however, of a role of miRNAs in this process, a recent bioinformatics study shows that presynaptic transcripts have an unusually long 3’UTR with an increased number of predicted miRNA target sites [21]. In addition, miR-1000 and the miR-310 cluster target presynaptic components and regulate neurotransmitter release in flies [22, 23].

We have previously identified miR-27b in a targeted screen for miRNAs that regulate presynaptic assembly in rodent cortical neurons [24]. miR-27b is highly-expressed in the brain [5] and has been associated with bipolar disorders and schizophrenia [25]. Here, we explore the impact of miR-27b on ~200 transcripts that make up the presynaptic compartment in excitatory neurons. We find, unexpectedly, that miR-27b up-regulates the expression of more than half of these presynaptic transcripts. This enhancing effect is due to miR-27b-dependent down-regulation of three main transcriptional repressors—Bmi1, Sox11 and Zfp90—that operate in concert to silence the presynaptic transcriptome. Our results further indicate that miR-27b-dependent silencing of Bmi1 shapes neural activity in cortical networks and suggest that posttranscriptional repression of Bmi1 is required for the developmental transition of a neural stem cell into a mature, synaptically-competent neuron.

## Results

### miR-27b boosts expression of presynaptic genes

**Fig. 1:**
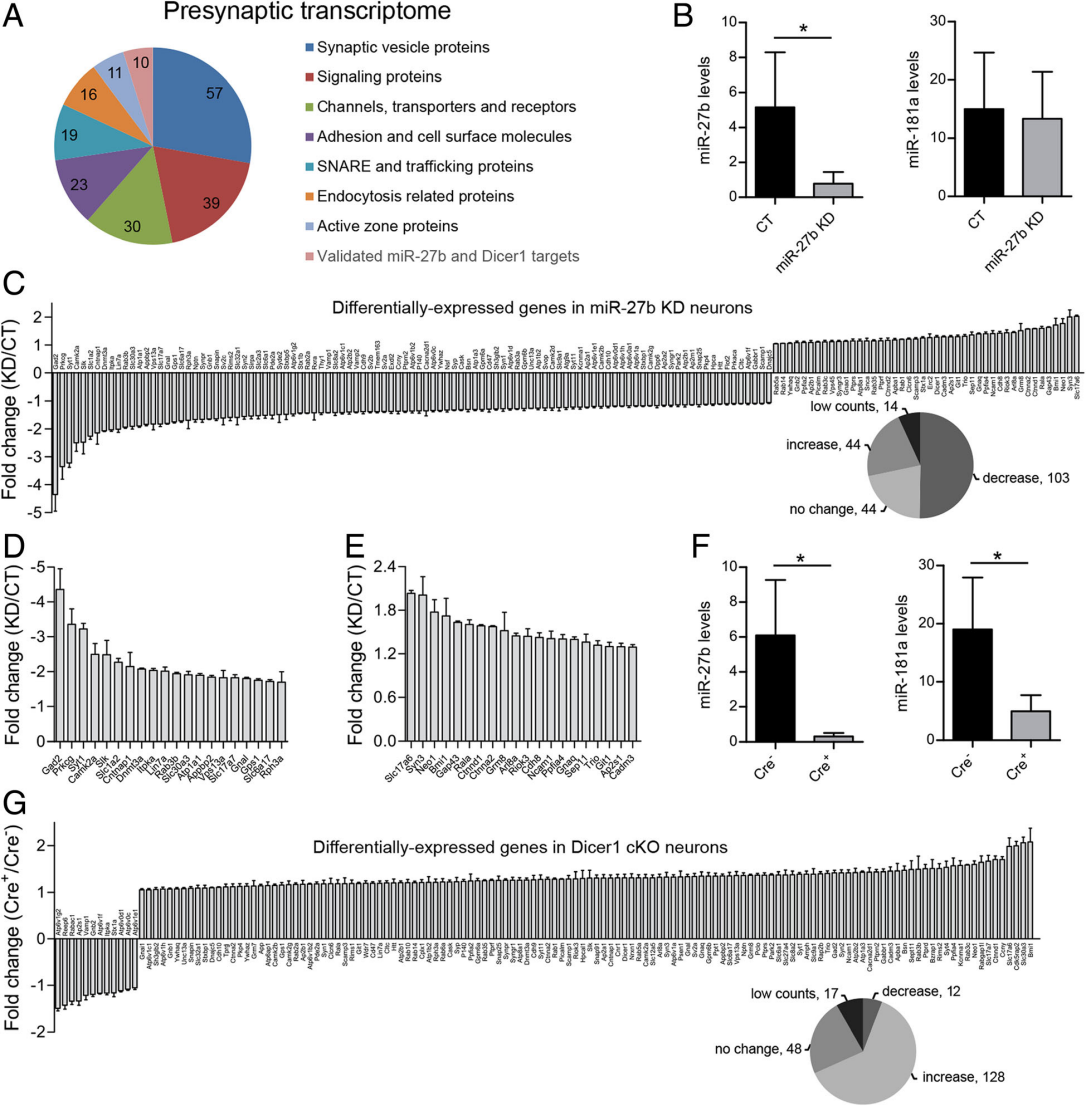
miR-27b KD globally alters expression of the presynaptic transcriptome. **a** Ontology-based classification of 195 presynaptic genes targeted by nCounter probes. nCounter probes were also designed against a few validated miR-27b and Dicer1 targets. **b** miRZip-27b (but not a control shRNA) suppressed miR-27b, but not miR-181a in DIV14 mouse cortical neurons (*n* = 3, * *p* < 0.05, *t*-test). miRNA levels were measured by qPCR. **c** nCounter profiling of the presynaptic transcriptome in miR-27b KD and CT neurons (DIV14). Genes were ranked based on their differential expression. Only genes that were significantly up- or down-regulated (147 out of 205) are shown (*n* = 3, *p* < 0.05, *t*-test). The pie chart indicates the number of genes up- and down-regulated, unchanged, or with low counts (discarded). **d**,**e** Top 20 most down- and up-regulated genes. **f** miR-27b and miR-181a levels probed by qPCR in *Dicer1* ^*fl/fl*^ cortical neurons (DIV14) transduced with a control (Cre^-^) or a Cre-expressing lentivirus (Cre^+^) (*n* = 3, * *p* < 0.05, *t*-test). **g** nCounter profiling of differentially-expressed genes in Cre^+^ vs Cre^-^ *Dicer1* ^*fl/fl*^ neurons. Only genes that were significantly up- or down-regulated are shown (*n* = 3, *p* < 0.05, *t*-test). Error bars indicate SD. See also Additional file 2: Table S2

To explore the global impact of miR-27b on genes associated with presynaptic function, we first compiled a list of 195 “presynaptic” genes, based on previous proteomics and functional studies (see Methods). These genes encode for the vast majority of proteins that make up the presynaptic compartment, and include synaptic vesicle (SV) proteins, active zone proteins, channels, adhesion molecules, trafficking and signaling proteins (Fig. 1a; Additional file 1: Table S1). We have also included in that list a handful of neuronally expressed and validated miR-27b and Dicer1 targets, many of which regulate gene expression (Additional file 1: Table S1). To knock down miR-27b in cortical neurons, we targeted miR-27b-3p—the guide strand of the miR-27b duplex and one of the most abundant miRNAs loaded onto the RNA-induced silencing complex (RISC) in the brain [6]—using a shRNA (miRZip-27b) cloned into a GFP-expressing lentiviral vector. Transduction of cortical neurons with miRZip-27b led to a specific and substantial reduction of miR-27b-3p levels (referred to hereafter as miR-27b) whereas a scrambled shRNA sequence (miRZip-Scr) had no effect (Fig. 1b). Because mammalian miRNAs silence gene expression predominantly by promoting message destruction [2], we profiled the mRNAs of these ~200 genes both in miR-27b knockdown (KD) and control (CT) neurons. As miRNAs typically have mild effects on the expression of most genes, we used a profiling approach (nCounter, Nanostring) that offers high levels of precision and sensitivity [26] and which is based on color-coded, gene-specific barcodes and multiplex single-molecule imaging of individual transcripts.

Analysis of differential gene expression showed that miR-27b significantly regulates 147 genes, which amounts to 76 % of the presynaptic transcriptome after the exclusion of 14 genes with low counts (Fig. 1c-e; Additional file 2: Table S2A). To our surprise, 70 % of the affected genes were down-regulated implying a positive influence of miR-27b on gene expression. To determine whether this type of regulation is unique to miR-27b, we profiled these transcripts in cortical neurons derived from a mouse homozygous for the floxed *Dicer1* allele (*Dicer1* ^*fl/fl*^ *)* and transduced with the Cre recombinase (or a control vector). We reasoned that if most miRNAs repress presynaptic genes, we should see an overall increase in gene expression in cells devoid of miRNAs. Transduction of Cre in *Dicer1* ^*fl/fl*^ neurons led to marked reduction of miR-27b and miR-181a, consistent with efficient Cre-mediated excision of the *Dicer1* gene (Fig. 1f). Among the 140 genes that are differentially regulated in *Dicer1* cKO neurons, 128 (91 %) were up-regulated, in agreement with a global negative impact of miRNAs on gene expression (Fig. 1g; Additional file 2: Table S2B). Thus, in contrast to the bulk of miRNAs, miR-27b exerts a positive influence on the presynaptic transcriptome.

### The transcriptional regulators Bmi1, Sox11 and Zfp90 are direct targets of miR-27b

**Fig. 2:**
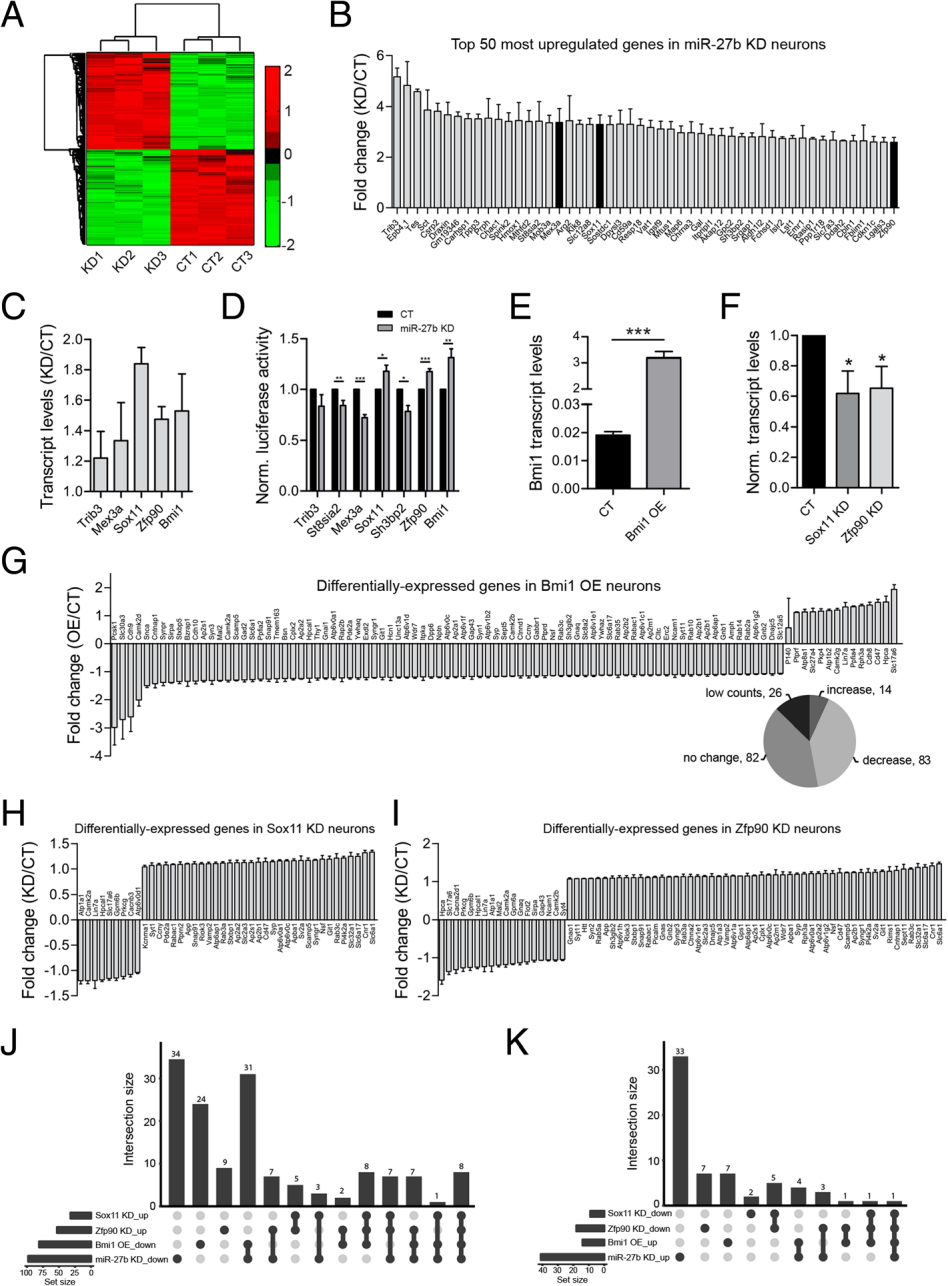
Sox11, Zfp90 and Bmi1 are miR-27b targets. **a**,**b** Genome-wide transcriptome analysis of miR-27b KD and CT mouse cortical neurons (DIV14). **a** Hierarchical clustering of differentially-expressed genes with fold-change ≥ 1.5 (FDR < 0.05). Columns represent individual experiments (KD, miR-27b knockdown; CT, miR-27b control). Rows correspond to individual genes. (See also Additional file 3: Table S3). **b** Top 50 most up-regulated genes in miR-27b KD neurons (*n* = 3, FDR < 0.006). Dark grey bars refer to candidate repressor genes. **c** qPCR analysis of Trib3 (the most-upregulated gene in the microarray data) and candidate repressors in miR-27b KD vs CT neurons (*n* = 4). Results are expressed as the KD/CT ratio. **d** Luciferase reporters containing the 3’UTR of candidate repressors (and a few other genes listed in (**b**)) were electroporated in neurons later transduced with miRZip-27b and miRZip-Scr lentiviruses. Luciferase activities were normalized to CT values (set to 1). (Trib3 and St8sia2, *n* = 5; Mex3a, *n* =6; Sox11 and Zfp90, *n* = 7; Sh3bp2, *n* = 4; Bmi1, *n* = 9. * *p* < 0.05, ** *p* < 0.01, *** *p* < 0.001, *t*-test). **e** qPCR analysis of Bmi1 expression in neurons (DIV14) transduced with a control vector or a Bmi1-expressing construct (*n* = 4, *** *p* < 0.001, *t*-test). **f** RNAi KD of Sox11 and Zfp90 in cortical neurons assessed by qPCR (*n* = 3, * *p* < 0.05, *t*-test). **g** nCounter profiling of the presynaptic transcriptome in CT and Bmi1 OE cortical neurons (*n* = 4, *p* < 0.05, *t*-test). 97 genes are differentially regulated. **h**,**i** nCounter analysis of the presynaptic transcriptome in Sox11- and Zfp90-silenced cortical neurons (*n* = 3, *p* < 0.05). 42 and 71 genes are differentially-expressed respectively. Error bars are SD except for C-E where they represent SEM. **j**,**k** Combinatorial intersection analysis of differentially-expressed genes in miR-27b KD, Bmi1 OE, Sox11- and Zfp90 KD neurons. **j** Intersections among genes repressed by Bmi1, Sox11 and Zfp90 and activated by miR-27b. **k** Intersections among genes repressed by miR-27b and activated by Bmi1, Sox11 and Zfp90

We hypothesized that the stimulatory effect of miR-27b on gene expression is indirect and is mediated by miR-27b-dependent silencing of transcriptional repressors or mRNA-destabilizing genes. To systematically search for putative miR-27b targets, we first analyzed the impact of miR-27b on gene expression at the genome-wide level using a microarray approach. Differential gene expression analysis revealed a total of 860 and 851 genes that were up- and down-regulated, respectively (fold-change > 1.5, false discovery rate < 0.05, Fig. 2a; Additional file 3: Table S3). Comparative analysis of the presynaptic transcriptome in the nCounter and microarray datasets reveals a 50 % overlap among the top 40 most differentially-expressed genes (Additional file 4: Table S4). Preferential down-regulation of presynaptic genes in miR-27b KD neurons is also observed in the microarray dataset (Additional file 5: Figure S1), but disappears at the genome-wide level (Fig. 2a; Additional file 3: Table S3), suggesting the stimulating activity of miR-27b only applies to a targeted subset of genes.

Next, we searched for candidate genes based on the extent of their up-regulation in miR-27b KD neurons, the presence of predicted miR-27b-binding sites in their 3’-UTR, and their reported function as transcriptional or post-transcriptional regulators. Three genes, *Mex3a*, *Sox11* and *Zfp90* were selected based on these criteria (Fig. 2b). We added to that list *Bmi1*, the 4th most upregulated gene in the nCounter dataset (Fig. 1e) and a validated miR-27b target [27]. We confirmed by qPCR that all four genes are upregulated in miR-27b KD neurons (Fig. 2c), and tested the ability of miR-27b to downregulate luciferase reporters containing the 3’-UTRs of these putative repressors. Reporters with the 3’-UTRs of *Bmi1*, *Sox11* and *Zfp90*, are up-regulated in miR-27b KD neurons (Fig. 2d), indicating that these three genes are likely targets of miR-27b.

### miR-27b turns on the presynaptic transcriptome by silencing Bmi1, Sox11 and Zfp90

Bmi1 is a polycomb group protein that functions as an epigenetic repressor [28] and maintains self-renewal of neural stem cells [29]. Sox11 is a member of the SoxC family of transcription factors that regulates neurogenesis in both the central and peripheral nervous system [30–32]. *De novo* Sox11 mutations cause Coffin-Siris syndrome, a congenital disorder characterized by microcephaly and intellectual disability [33]. Less is known about the zinc-finger protein Zfp90. This transcription factor regulates the transcriptional repressor REST [34] and is located in a 16q22.1 microdeletion in a family with mental retardation [35].

To determine which, if any, of these three transcriptional regulators mediate miR-27b’s effect on the presynaptic transcriptome, we profiled presynaptic transcripts in neurons with altered levels of Bmi1, Sox11 and Zfp90. Each of these three transcriptional regulators functioned, globally, as a repressor (Fig. 2g-i). Overexpression of Bmi1 (Fig. 2e) resulted in repression of 81 genes, 47 of which (58 %) overlapped with miR-27b up-regulated genes (Fig. 2j). Only a dozen genes were upregulated by Bmi1, with little intersection (5 out of 41, 12.2 %) with miR-27b repressed genes (Fig. 2k). The striking similarity between Bmi1-repressed and miR-27b up-regulated genes suggests that Bmi1 is a major functional target of miR-27b.

Because we were unable to overexpress Sox11 and Zfp90 at sufficient levels (not shown), we silenced them, instead, by lentiviral delivery of shRNAs. This approach resulted in a moderate (~40 %) reduction of Sox11 and Zfp90 transcripts (Fig. 2f). Fewer presynaptic genes are significantly regulated by Sox11 or Zfp90 (Fig. 2h,i), which could reflect incomplete knockdown of these transcriptional regulators. Among the genes repressed by Sox11 and Zfp90 (i.e. up-regulated in Sox11- and Zfp90-silenced neurons), 19 (59 %) and 29 (55 %) intersected, respectively, with miR-27b upregulated genes (Fig. 2j). More than 47 % of Sox11- and Zfp90-repressed genes were co-repressed by Bmi1, while Sox11 and Zfp90 shared 87 % of their down-regulated target genes (Fig. 2j). Only a handful of genes were up-regulated by Sox11 and Zfp90 (Fig. 2h,i) with little overlap with miR-27b repressed, or Bmi1 stimulated genes (Fig. 2k). Thus, there is significant overlap in genes repressed by Bmi1, Sox11 and Zfp90 and silencing of these three transcriptional regulators by miR-27b likely has an additive effect on the expression of presynaptic genes. These data led us to infer that miR-27b shapes the presynaptic transcriptome by silencing at least three transcriptional repressors with intersecting target genes.

### Gene set enrichment analysis predicts roles of miR-27b in synaptic transmission and repression of a proliferative state

We were intrigued by the fact that two out of three repressors of the presynaptic transcriptome—Bmi1 and Sox11—are central regulators of neural stem cell renewal and neurogenesis. This led us to speculate that miR-27b functions as a genetic switch by suppressing proliferation and promoting synapse maturation.

**Fig. 3:**
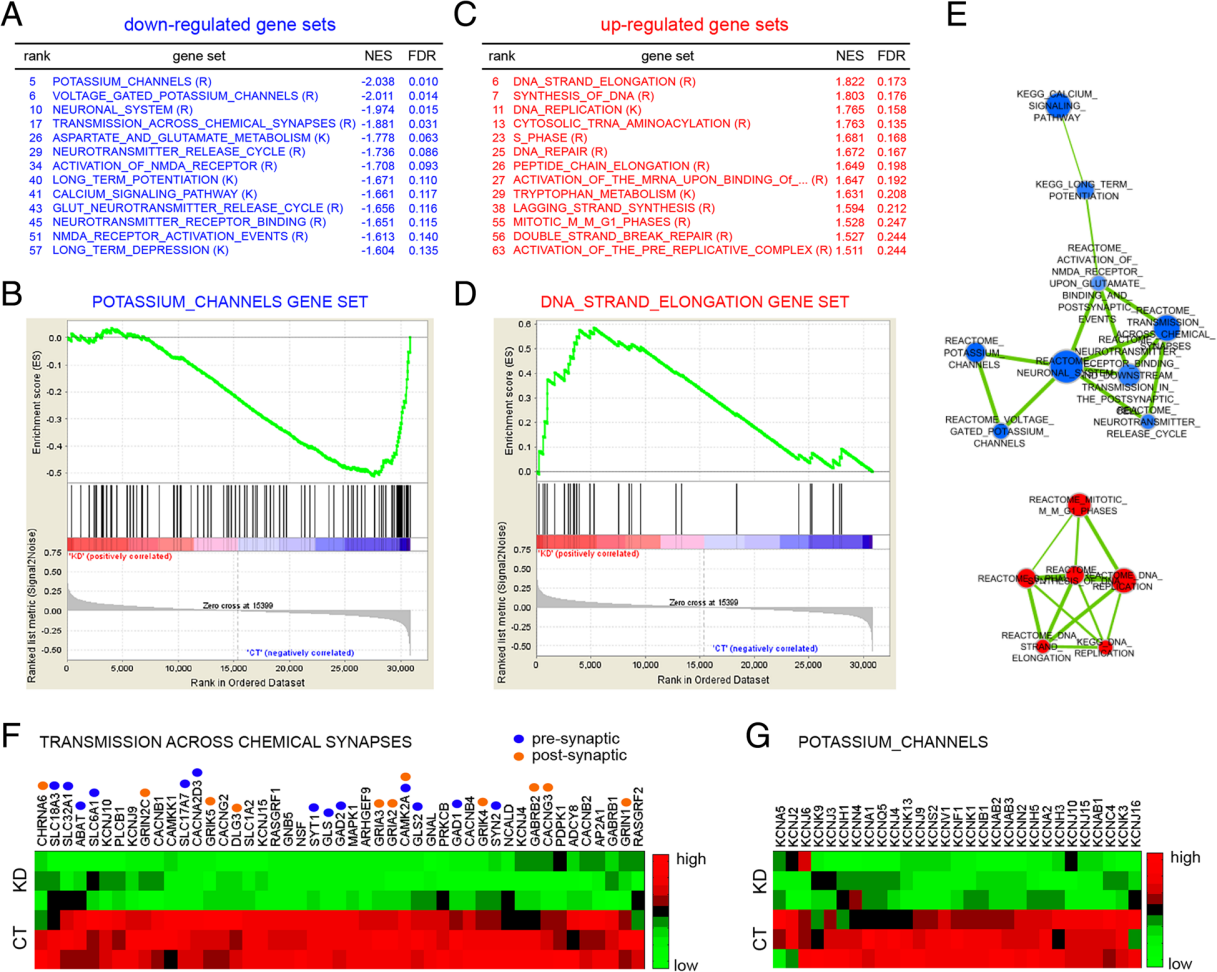
Gene set enrichment analysis (GSEA) identifies miR-27b-regulated gene networks associated with neurotransmission and cell proliferation. **a**-**d** GSEA analysis of the microarray data. Out of a total of 1330 gene sets, 63 were significantly enriched at the top and 113 were significantly enriched at the bottom of the gene ranked list (See Additional files 6 and 7: Tables S5,S6). **a**,**c** Selected down- and up-regulated gene sets linked to neurotransmission (**a**) and cell proliferation (**c**). Gene sets are ranked according to their normalized enrichment score (NES). The false discovery rate (FDR) is the estimated probability that a gene set with a given NES represents a false-positive. K (KEGG) and R (Reactome) indicate the source of pathways. **b**,**d** Enrichment score (ES) plots for the indicated gene sets. Negative and positive ES values point to gene sets over-represented in the top most down- or up-regulated genes in miR-27b KD neurons. Vertical bars refer to individual genes in a gene set and their position reflects the contribution of each gene to the ES. Genes that belong to the leading edge subset (i.e. genes that appear at or after the ES minimum (**b**) and at or before the ES maximum (**d**)) contribute to the enrichment signal. **e** Network analysis of gene sets. Shown are the two largest networks of down-regulated (*blue*) and up-regulated (*red*) gene sets. They are comprised of gene sets listed in (**a**) and (**c**) (see Additional file 8: Figure S2 for the visualization of all detected networks). Node size is proportional to the number of genes in the microarray that are represented in the gene set. Color intensity of the node (*light to dark*) indicates the significance of the gene set. Edge size corresponds to the number of genes that overlap between two connected gene sets. **f**,**g** Hierarchical clustering of differentially-expressed genes belonging to the leading edge subset of the indicated gene sets. Rows indicate individual miR-27b CT and KD conditions. **f** Genes with clear roles in pre- or post-synaptic functions are indicated

To test this hypothesis further, we performed a gene set enrichment analysis (GSEA) [36, 37] on the microarray data described earlier, to find out whether gene sets related to synaptic transmission and cell proliferation are systematically altered in miR-27b KD neurons. We first ranked all genes according to the extent of their differential expression in miR-27b KD and CT cells. We then computed normalized enrichment scores (NES) for a collection of 1330 curated gene sets representing canonical biological pathways, and identified gene sets that are overrepresented at both extremes of the ranked list. Among the top 60 gene sets associated with down-regulated genes in miR-27b KD neurons, 13 of them (22 %) were related to neuronal excitability, synaptic transmission, neurotransmitter release and synaptic plasticity (Fig. 3a, b; Additional file 6: Table S5). At the other extreme, up-regulated genes belonged to gene sets (12 out of the top 60, 20 %) associated with DNA synthesis and the cell cycle (Fig. 3c, d; Additional file 7: Table S6). To functionally visualize these enrichment results, we displayed them as a network where pathways are nodes and edges represent pathway crosstalk [38]. This analysis revealed two main functional gene modules related to synaptic transmission and DNA synthesis (Fig. 3e and Additional file 8: Figure S2). Together, these data show that miR-27b reciprocally regulates gene networks associated with neurotransmission and cell proliferation.

To identify miR-27b-regulated genes potentially involved in neurotransmission, we extracted core members of the transmission-across-chemical-synapse gene set (Fig. 3a) that contribute to the enrichment score. Hierarchical clustering of these genes shows, as expected, that they are significantly down-regulated in miR-27b KD neurons (Fig. 3f). Many of these genes have clear presynaptic functions and five of them, *Gad2* (GAD65, Glutamate Decarboxylase 2)*, Syt1* (Synaptotagmin 1)*, Slc17a7* (VGLUT1, Vesicular Glutamate Transporter)*, Slc32a1* (VGAT, Vesicular GABA Transporter) and *Syn2* (Synapsin II) belong to the top 30 most down-regulated genes in the nCounter dataset (Fig. 1c; Additional file 2: Table S2). Of interest, these genes include regulators of both glutamate and GABA metabolism and transport, pointing to a role of miR-27b in both excitatory and inhibitory neurotransmission. A similar number of genes encode for postsynaptic proteins (Fig. 3f), including AMPA-receptor (*Gria2* and *Gria3*), NMDA-receptor (*Grin1* and *Grin2c*) and kainate-receptor (*Grik4*) subunits, suggesting both pre- and post-synaptic functions for miR-27b. Moreover, the positive impact of miR-27b on the expression of 29 potassium channels (Fig. 3g), most of which are voltage-gated, hints at a broader role of miR-27b in neuronal excitability.

### miR-27b stimulates neural network activity by repressing Bmi1

**Fig. 4:**
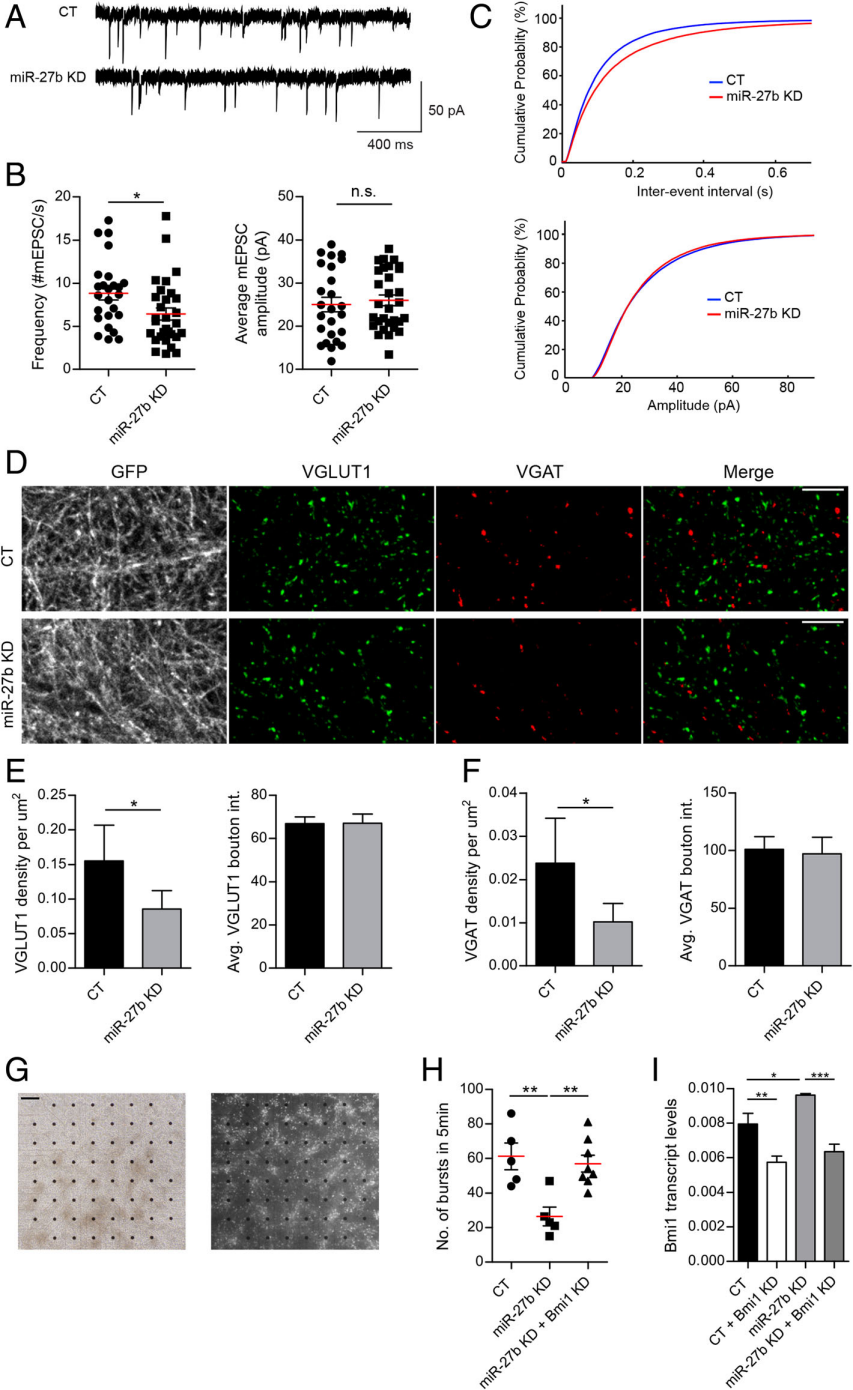
miR-27b enhances neurotransmission. **a**-**c** Whole-cell patch clamp recordings of mEPSCs in miR-27b KD and CT hippocampal neurons (DIV14). **a** Representative examples of individual mEPSC traces for both conditions. **b** Quantification of mEPSC frequency and amplitude in miR-27b CT (*n* = 25) and KD (*n* = 30). * *p* < 0.05, *t*-test. Red bars indicate the Mean and error bars represent SEM. Each symbol corresponds to data from a single neuron. **c** Cumulative distributions of mEPSC frequency and amplitude. **d**-**f** Immunostaining of VGLUT1 and VGAT in miR-27b KD and CT neurons. Scale bar: 10um. **e**,**f** Quantification of puncta density and intensity for (**e**) VGLUT1 (16 fields) and (**f**) VGAT (16 fields) from 3 experiments. *p < 0.05, *t*-test. **g**-**i** MEA recordings of miR-27b CT and KD cortical neurons (DIV15), or miR-27b KD neurons transduced with a Bmi1-targeting shRNA. **g** Bright-field (*left*) and mCherry fluorescence (*right*) images of a neuron network grown on an MEA chip. mCherry reports expression of miRZip-27b. Scale bar: 200um. **h** Quantification of burst frequency for the indicated conditions. Each symbol represents bursting activity of a single MEA culture dish. MEA dishes originate from three independent experiments. Red bars indicate the Mean (** *p* < 0.01, one-way ANOVA). **i** qPCR detection of Bmi1 transcripts in cortical neurons expressing miRZip-Scr, Bmi1-shRNA, miRZip-27b and miRZip-27b + Bmi1-shRNA (*n* = 7, * *p* < 0.05, ** *p* < 0.01, *** *p* < 0.001, one-way ANOVA). Error bars represent SEM

These GSEA results prompted us to examine the effect of miR-27b on synaptic transmission. We first measured miniature excitatory post-synaptic currents (mEPSCs) in miR-27b KD and CT neurons by whole-cell patch clamp recordings. mEPSCs are driven by spontaneous release of glutamate from excitatory inputs, in the absence of action potential (AP) firing. The frequency of mEPSCs, but not their amplitude, was markedly decreased in miR-27b KD neurons (Fig. 4a-c). While mEPSC amplitude is governed by the number and/or activity of AMPARs on the post-synaptic membrane, mEPSC frequency depends on the probability of neurotransmitter release and the number of excitatory inputs on the recorded neuron. To find out whether miR-27b regulates the formation of excitatory synapses, we immunostained vGLUT1, a marker of excitatory synaptic vesicles, in miR-27b KD and CT neurons. Because miR-27b also boosts expression of genes linked to GABA neurotransmission (Figs. 1d and 3f) we also labeled the SV-resident GABA transporter VGAT in these cells. As anticipated, both SV markers label distinct populations of boutons and excitatory synapses far exceed inhibitory synapses in these neuron cultures (Fig. 4d). A quantitative analysis of these puncta shows a marked reduction (about 50 %) in both glutamatergic and GABAergic terminals (Fig. 4d-f). Surprisingly, however, the average intensity of VGLUT1 and VGAT puncta is unaffected by miR-27b knockdown, despite significant downregulation of VGLUT and VGAT transcripts in miR-27b KD neurons (Fig. 3f; Additional file 2: Table S2A), suggesting that miR-27b preferentially regulates the number of synapses rather than the abundance of these SV markers at synaptic terminals. The stimulatory effect of miR-27b on synaptogenesis was further confirmed using the active zone scaffold bassoon as a presynaptic marker (Additional file 9: Figure S3). Therefore, reduced number of excitatory synaptic inputs likely contributes to decrease in mEPSC frequency in miR-27b KD neurons.

To evaluate the impact of miR-27b on neuronal excitability and AP firing, we measured spontaneous network activity in miR-27b KD and CT cortical neuron cultures using multi-electrode arrays (MEAs). After 2 weeks in vitro, these neuronal networks exhibit spontaneous activity consisting of single spikes and bursts that can be recorded in real-time and non-invasively on an MEA chip (Fig. 4g). Control cultures display an average of 61.3 ± 7.6 SEM bursts in 5 min (Fig. 4h). Average burst frequency was markedly reduced, however, in miR-27b KD neurons (26.5 ± 5.5 SEM). To determine whether miR-27b boosts network activity by repressing Bmi1, we co-silenced miR-27b and Bmi1 by transducing neurons with both miRZip-27b and a Bmi1-targeting shRNA, in an attempt to restore network activity. We verified first that Bmi1 levels are efficiently brought down in miR-27b KD neurons by knockdown of Bmi1 (Fig. 4i). Neuron cultures co-expressing miRZip-27b and a Bmi1 shRNA fire bursts at a frequency similar to control cells (57.1 ± 4.8 SEM), providing evidence for a role of Bmi1 in miR-27b-dependent regulation of network activity (Fig. 4h). Collectively, these data show that miR-27b boosts neurotransmission by acting, at least in part, on the presynaptic compartment and suggest that de-repression of the presynaptic transcriptome by miR-27b silencing of Bmi1 is sufficient to support efficient neurotransmission in neural networks.

## Discussion

Previous analyses of miRNA-mRNA networks have suggested that miRNAs function primarily as rheostats—they buffer gene expression but rarely act as master regulators [39, 40]. miR-27b appears to be an exception to this rule. By silencing three transcriptional repressors that target overlapping sets of genes, miR-27b has a profound impact on the presynaptic transcriptome. More than half (54 %) of all genes that make up the presynapse are positively influenced by miR-27b. The stimulatory effect of miR-27b on presynaptic genes is greater than its silencing activity, both in terms of magnitude (i.e fold-change in gene expression) and number of genes affected. This suggests the presence of an underlying amplification mechanism, which, we propose, is mediated by the cooperative repressive activity of three miR-27b targets. The high fraction of genes co-regulated by miR-27b and Bmi1 (58 %) suggest that silencing of this epigenetic repressor significantly contributes to miR-27b-dependent up-regulation of presynaptic genes. Rescue of network activity in miR-27b-silenced cortical neurons by RNAi KD of Bmi1 supports a functional role of this epigenetic repressor downstream of miR-27b. Because most genes repressed by Sox11 and Zfp90 overlap with those targeted by Bmi1, there remains about 40 % of miR-27b up-regulated genes that could potentially be under the control of other repressors.

Bmi1 is a core component of the Polycomb Repressive Complex 1 (PRC1), which, together with PRC2, is widely deployed in eukaryotic cells to implement gene silencing [41]. Bmi1 drives self-renewal of neuronal stem cells in the forebrain and cerebellum by suppression of cell cycle inhibitors [42, 43]. Our data now show that Bmi1 also represses a significant fraction of genes associated with presynaptic functions, suggesting a novel mode of action of this epigenetic regulator in maintaining an undifferentiated state. By promoting cell proliferation and repressing genes implicated in neurotransmission, Bmi1 puts a two-tier break on cell differentiation. We propose that miR-27b-directed silencing of Bmi1 (and other repressors such as Sox11 and Zfp90) relieves this break and drives (pre)synaptic maturation of excitatory neurons. This model is supported by our GSEA results, which identify neurotransmission and cell proliferation as the largest and most significant gene networks reciprocally regulated by miR-27b. In this context, it is worth mentioning the opposite effect of miR-27b on VGLUT1 (*Slc17a7*) and VGLUT2 (*Slc17a6*) expression—VGLUT1 is down-regulated 1.8-fold, while VGLUT2 is the most up-regulated presynaptic gene in miR-27b KD neurons (nCounter dataset). A clear reciprocal expression of these two vesicular glutamate transporters is also observed in the microarray data set. Of note, VGLUT1 and VGLUT2 mark distinct, largely non-overlapping populations of glutamatergic neurons in the CNS—VGLUT1 is expressed in the neocortex (layers I-III), hippocampus and amygdala, while VGLUT2 is found in layer IV of the cerebral cortex, dentate gyrus, thalamus and hypothalamus [44]. This suggests the possibility that miR-27b promotes differentiation of specific, VGLUT1-positive, lineages of excitatory neurons. Strikingly, VGLUT2 is also the most up-regulated gene in Bmi1-overexpressing cells, indicating that its repression in cortical neurons may also occur through miR-27b-directed silencing of Bmi1. The stimulatory effect of miR-27b on synapse development is not, however, restricted to excitatory neurons. Knockdown of miR-27b also results in downregulation of GABA specific genes and in reduced density of inhibitory synaptic terminals, suggesting wider functions of miR-27b in excitatory and inhibitory balance.

Given the broad impact of miR-27b on the presynaptic transcriptome, and its influence on post-synaptic and neural excitability genes, it is unlikely that miR-27b’s effect on neuronal activity is dominated by any one gene (other than transcriptional repressors). Nevertheless, miR-27b turns on or off several key genes implicated in synaptic transmission. Perhaps the most obvious candidate is the calcium sensor synaptotagmin-1 (*Syt1*), an abundant integral protein targeted to SVs that controls both evoked and spontaneous neurotransmitter release [45]. miR-27b elevates *Syt1* expression by more than 2-fold in both the microarray and nCounter datasets. *Syt1* is ranked third among down-regulated presynaptic genes and is within the top 0.2 % most down-regulated genes (genome-wide) in miR-27b KD neurons. It is also substantially repressed by Bmi1. Because *Syt1* is required for evoked, synchronous neurotransmitter release [46, 47], it is possible that its down-regulation in miR-27b KD neurons contributes to reduced network activity. It is improbable, however, that *Syt1* plays a major role in miR-27b-dependent regulation of mEPSC frequency, as Syt1 ablation results, instead, in an increase in mEPSC frequency [48], attributed to a clamp-like activity of *Syt1* in resting cells. Several other presynaptic genes, all up-regulated more than two-fold by miR-27b, are likely to contribute to this synaptic deficit. These include *Lin7a* (Veli/MALS), an active zone scaffold [49], *Slc17a7* (VGLUT1), recently implicated in coordination of SV cargo endocytosis [50], *Rims2* (RIM2) and *Syn2* (Synapsin II), which regulate vesicle docking at the active zone [51], and Snapin, a 15 kDa protein that accelerates SV recycling [52]. Collectively, both the genomics and neurophysiology data point to a global and positive influence of miR-27b on presynaptic function. It should be noted, however, that miR-27b also silences a handful (44) of presynaptic genes, including positive regulators of neurotransmitter release, such as Liprin-α4 (*Ppfia4)* and Syntaxin-1A (*Stx1A*). Thus, the impact of miR-27b on neurotransmission is complex and likely involves bidirectional regulation of multiple synaptic components.

## Conclusion

We describe in this paper a post-transcriptional circuitry that enables a single miRNA to exert considerable influence on the expression of functionally related genes. This network is based on miRNA-directed silencing of transcriptional repressors with overlapping, ontology-related targets, and departs from current models of miRNA-gene interactions, which ascribe to miRNAs a widespread, but modest effect on gene expression [53]. This circuitry may be well suited to drive irreversible cellular responses, such as cell differentiation.

## Methods

### Mouse strains, DNA constructs and lentiviruses

*Dicer1* ^*fl/fl*^ mice were first described in [54] and were obtained from Jackson Laboratory (*Dicer1* ^*tm1Bdh*^, #006366). The primers used for genotyping are described in [54]. miRZip-Scr (Cat: MZIP000-PA-1) and miRZip-27b (Cat: MZIP27b-PA-1) were purchased from Systems Biosciences (SBI). The anti-miR-27b shRNA targets both mouse and rat miR-27b-3p. The small hairpin sequence targeting miR-27b and its corresponding control (Scr) were subcloned in a modified pFUGW lentiviral vector containing an additional U6 promotor to drive expression of shRNAs (pFU6UGW). In some experiments, we used a red version of these constructs by replacing GFP with mCherry. The Cre-IRES-GFP construct was a gift from Dr. S. Je (Duke-NUS Medical School, Singapore). Cre-IRES-GFP was subcloned into a modified pFUGW lentiviral vector that lacks GFP. Mouse Bmi1-IRES-GFP (#21577) and Bmi1-shRNA (#21576) were obtained from Addgene. Both constructs were made in a pFUGW backbone vector. Lentiviral particles based on the pFUGW vector were generated as previously described [24]. A multiplicity of infection (MOI) between two and five was used for all viral transduction experiments. Lentiviral particles expressing shRNAs against mouse Sox11 (Cat: V3SR7601-10EG498945) and Zfp90 (Cat: V3SR7601-10EG84046) were purchased from Dharmacon. Luciferase sensors were generated by PCR amplification and cloning of 3’UTRs into psiCHECK2 [55]. All oligos used in this study are listed in Additional file 10: Table S7 and all DNA constructs were sequenced before use. Antibodies against VGLUT1 (MAB5502, Chemicon), VGAT (131003, SySy) and Bassoon (ab82958, Abcam) were used for immunofluorescence studies.

### Primary mouse and rat neuron cultures

Mouse cortical neurons were isolated from embryonic E16 *Dicer1* ^*fl/fl*^ mouse embryos and dissociated with papain as described previously [56]. 500,000 neurons were seeded on 12-well poly-L-lysine-coated tissue culture dishes. Neurons were transduced with lentiviruses 3 h after plating and cultured in Neurobasal media with B27 supplement. For nCounter analysis, cell lysates were prepared from DIV14 neurons using 150 μl of RLT buffer (Qiagen) per well. RNA was also extracted from the same three neuronal preparations for qPCR analysis using Sepasol RNA I Super G. For microarray analysis, RNA was isolated from DIV14 neurons using the RNeasy Mini kit (Qiagen) and RNA quality was analysed on a Bioanalyzer 2100 (Agilent Technologies). Rat cortical and hippocampal neurons were isolated from E18 rat embryos as previously described [24]. Lentiviruses were added 3 h or a day after plating. For electrophysiology recordings, 100,000 rat hippocampal neurons were seeded on 18 mm poly-L-lysine-coated glass coverslips and grown for 15–18 days before patching. For MEA recordings, 100,000 rat cortical neurons were seeded in MEA culture dishes and grown for 15 days before network activity was measured. For qPCR, RNA was extracted from 500,000 to 750,000 cortical neurons (DIV14) grown on poly-L-Lysine-coated 12-well tissue culture dish. For luciferase assays, 1 million cortical neurons were electroporated with luciferase sensors and seeded in poly-L-lysine-coated 24-well tissue culture plate and cell lysates were prepared after a week in culture.

### nCounter profiling of the presynaptic transcriptome

We compiled a list of 195 “presynaptic” genes from the following papers and reviews [21, 57–60]. This list consists of genes whose products have been identified through biochemical/proteomics approaches in pre-synapses (or sub-structures of presynaptic terminals), and/or have been functionally implicated in presynaptic functions. We added to that list known miR-27b targets: *Runx1* [61], *Rxra* [62] and *Bmi1* [27] and 6 reported *Dicer1* targets (*Appbp2, Tgfbr3, Ccny, Neo1, Exd2, Dnmt3a*) [63], four of which are also predicted miR-27b targets (*Appbp2, Tgfbr3, Ccny, and Neo1*). Seven genes were selected for signal normalization—4 ribosomal subunits (*Rpl24, Rpl30, Rps16, Rps20*), *Gapdh*, *Cript* and *Cnga2.* These normalization genes contain no predicted miRNA binding sites and have been defined by others as housekeeping genes in the CNS [64, 65]. A total of 212 nCounter probes were designed against these genes and synthesized by Nanostring Technologies (Seattle, WA). Accession numbers of these genes and probe sequences are listed in Additional file 1: Table S1. Hybridization and single-molecule detection of these probes were conducted on the nCounter multiplex platform at the Genome Institute of Singapore or in the S. Albani lab (SingHealth Translational Immunology and Inflammation Centre, Singapore). Probe counts were analysed with the nSolver software (Nanostring Technologies). We used the geometric mean of six normalization genes (*Cnga2* was discarded because of its low counts) as normalization factor. Triplicates were run, each corresponding to an independent neuron preparation with both miR-27b CT and KD conditions. We used log2-transformed fold-change (FC) to display differential gene expression: log2FC = log2(KD) –log2(CT); FC = 2 ^ log2FC. For combinatorial intersection analysis of nCounter datasets, we used the open source CRAN R package UpSet [66].

### Microarray profiling and Gene Set Enrichment Analysis (GSEA)

Microarray analysis was performed by the Duke-NUS Genome Biology Facility on an Illumina platform (San Diego, CA). Biotin-labeled cRNA was prepared using the TotalPrep RNA amplification Kit (Ambion) from 500 ng total RNA. 1.5 ug of biotin-labeled cRNA was hybridized to MouseWG-6 v2.0 expression beadchips, and chips were washed and stained according the manufacturer’s instructions. Beadchips were scanned using the Illumina BeadArray reader. Expression data were analysed using the R-Bioconductor open source software (limma R package) and are derived from triplicate experiments, each corresponding to one neuronal preparation with both miR-27b CT and KD conditions. Genes with fold-change > 1.5 and a false discovery rate < 0.05 were considered as differentially-regulated. Hierarchical clustering, dendrograms and heat maps were performed and displayed using the clustergram function in Matlab. For GSEA analysis of microarray data, we used the GSEA software available on the GSEA-Broad Institute website. We ran our expression dataset against a library of 1330 curated gene sets for canonical pathways (c2.cp.v5.1.symbols.gmt; MSigDB v 5.1). Qualitatively similar results were obtained with a gene ontology (GO) gene set library (c2.bp.v5.1.symbols.gmt). The statistical significance (nominal p value) of the enrichment score (ES) was estimated by running 1000 gene set permutations. The ES was normalized (NES) to account for the size of the gene set. To adjust for multiple testing across the 1330 gene sets, we computed the false discovery rate (FDR) and controlled the FDR at 25 %. Gene networks were visualized using the open source software platform Cytoscape 3.3.

### RT-qPCR and luciferase assays

For quantification of miRNA levels in mouse neurons, we used Taqman RT and qPCR primers (Applied Biosystems) specific for Y1 scRNA (control), miR-27b-3p, and miR-181a, as previously described [24] Y1 was used for normalization. For all other RT-qPCR experiments, RNA was extracted from DIV14 mouse or rat neurons using the Qiagen RNeasy Mini kit. qPCR primers used for mouse Trib3, Mex3a, Sox11, Zfp90, Bmi1, and rat Bmi1 are described in Additional file 10: Table S7. Reverse transcription was done using the iScript cDNA synthesis kit (BioRad). qPCR reactions were run in triplicates with iQ SYBR Green supermix (BioRad) and KiCqStart SYBR Green primers (Sigma). Reactions were performed on the Bio-Rad CFX-96 machine (BioRad) and actin was used for normalization. Luciferase assays were conducted using the Dual-Luciferase Reporter kit (Promega) as described previously [24]. Luciferase sensors tagged with the 3’UTR of rat Trib3, St8sia2, Mex3a, Sox11, Sh3bp2, Zfp90 and Bmi1 were introduced in rat cortical neurons by nucleofection (Amaxa, Lonza). After a week in culture, firefly and renilla luminescences were sequentially measured and Renilla counts (under 3’UTR control) were normalized to firefly counts.

### Whole-cell recordings and multi-electrodes arrays (MEAs)

Whole-cell patch-clamp recordings were performed in DIV15-18 hippocampal neurons, using a multi-Clamp 700B amplifier driven by the pClamp software (Axon Instruments, Sunnyvale, CA), as previously described [67]. Cells were held at -70 mV. Patch pipettes (3-5 MΩ resistance) were filled with an internal solution containing (in mM) 120 K-Gluconate, 9 KCl, 10 KOH, 3.48 MgCl_2_, 4 NaCl, 10 HEPES, 4 Na_2_ATP, 0.4 Na_3_GTP, 19.5 Sucrose, 5 EGTA. Neurons were bathed in external solution containing (in mM) 110 NaCl, 5 KCl, 2 CaCl_2_, 0.8 MgCl_2_,10 HEPES pH 7.4 and 10 D-glucose and supplemented with 0.5 μM TTX, to prevent action potential-evoked EPSCs, and 10 μM BMI to block GABAergic inhibitory postsynaptic potentials. Data were analyzed using pClamp. Only recording epochs in which series and input resistances varied by <10 % were included in the analysis. The identity of miR-27b CT and KD groups was concealed from the experimenter. For MEA recordings, rat cortical neurons were plated on culture dishes containing an array of 60 (8 x 8) electrodes (Multi-Channel Systems). Extracellular recordings were performed on an air-suspended table in a Faraday cage. Signals were amplified using the MEA1060 hardware system (Multi-Channel Systems), and recorded in real-time for 5 min using the MC_rack software (Multi-Channel Systems). High pass (300 Hz) and low pass (3 kHz) filters were applied to the data. Spikes were detected using a voltage threshold rule (standard deviation > 6). Data were transferred to Matlab to determine the frequency of population bursts using a custom-designed script.

### Immunocytochemistry and image analysis of SV puncta

Hippocampal neurons (DIV15) grown on glass coverslips were fixed in 4 % PFA, 4 % sucrose, permeabilized with 100 ug/ml digitonin for 10 min and blocked with 5 % goat serum for 1 h. Fixed cells were co-incubated with primary Abs against VGLUT1 (mouse Ab) and VGAT (rabbit Ab) and secondary Abs conjugated to Alexa Fluor 568 (anti-mouse IgG) or 633 (anti-rabbit IgG). Neurons were then imaged for GFP, VGLUT1 and VGAT using a laser scanning confocal microscope (LSM710, Zeiss). Puncta density and intensity was quantified using Matlab-based scripts described previously [24, 68].

### Statistical analysis

Statistical tests used for the genomics data (nCounter, microarray and GSEA) were described in previous sections and in figure legends. Average data are represented as means ± SD unless stated otherwise. Statistical significance was determined using two-tailed unpaired t-tests on data sets consisting of two groups. One-way ANOVA was used when simultaneously comparing three or more data sets. In this case, p values were derived from a post-hoc Bonferroni test.

## Additional files

Additional file 1: Table S1. List of nCounter probes used in this study. (XLSX 26 kb)
Additional file 2: Table S2. Differentially-expressed presynaptic genes in miR-27b KD (A) and *Dicer1* cKO (B) neurons. (nCounter dataset). (XLSX 73 kb)
Additional file 3: Table S3. Up (A) and Down (B) regulated genes in miR-27b KD neurons (microarray dataset). (XLSX 380 kb)
Additional file 4: Table S4. Comparative analysis of the presynaptic transcriptome in miR-27b KD neurons by nCounter or microarray profiling. (XLSX 11 kb)
Additional file 5: Figure S1. Microarray analysis of the presynaptic transcriptome in miR-27 KD (*n* = 3) and CT (*n* = 3) neurons. Shown are differentially-expressed genes (FDR < 0.05). (TIF 235 kb)
Additional file 6: Table S5. List of down-regulated gene sets in miR-27 KD neurons. (XLSX 21 kb)
Additional file 7: Table S6. List of up-regulated gene sets in miR-27 KD neurons. (XLSX 18 kb)
Additional file 8: Figure S2. Gene networks visualized using Cytoscape3.3 and generated from both up- (red) and down- (blue) regulated gene sets in miR-27 KD neurons. (TIF 1178 kb)
Additional file 9: Figure S3. Bassoon immunostaining in miR-27 KD and CT hippocampal neurons. (TIF 765 kb)
Additional file 10: Table S7. List of DNA primers used in this study. (XLSX 11 kb)
