# Supplementary figures and images for: miR-27b shapes the presynaptic transcriptome and influences neurotransmission by silencing the polycomb group protein Bmi1
Source: BMC Genomics. 2016 Oct 4;17:777. doi: 10.1186/s12864-016-3139-7 (PMC5050705; doi:10.1186/s12864-016-3139-7)

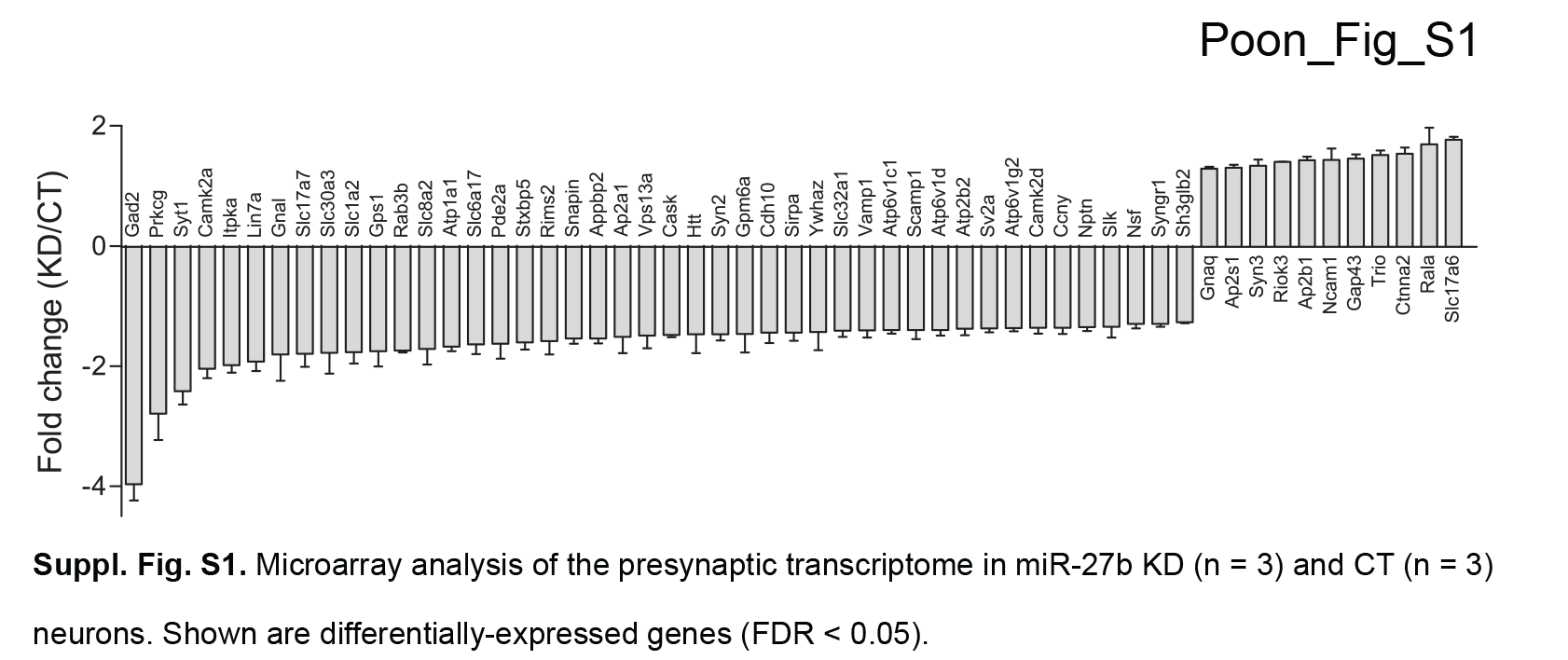

Supplement: Additional file 5: Figure S1. — Microarray analysis of the presynaptic transcriptome in miR-27 KD (n = 3) and CT (n = 3) neurons. Shown are differentially-expressed genes (FDR < 0.05). (TIF 235 kb) [file 12864_2016_3139_MOESM5_ESM.tif]

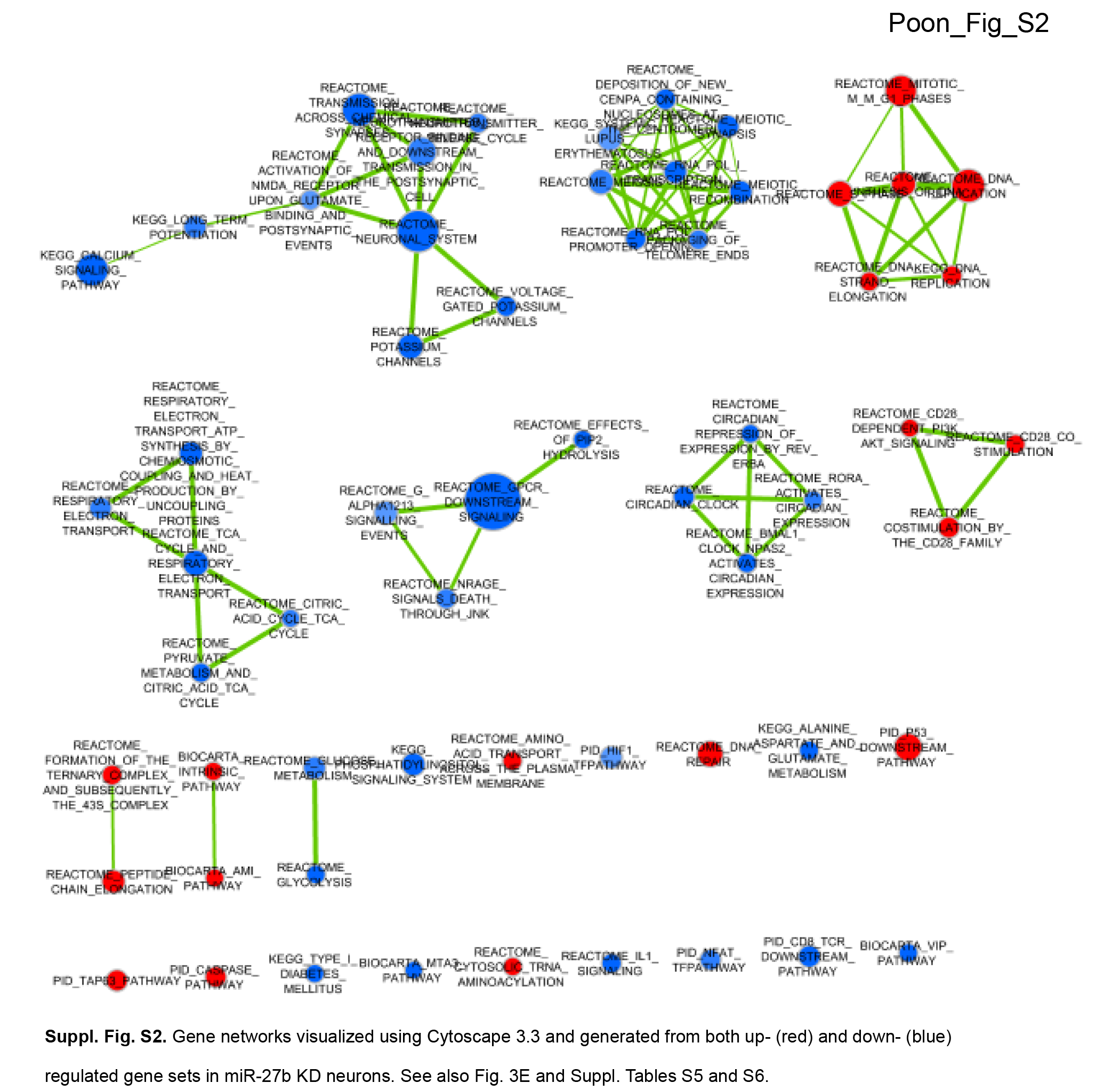

Supplement: Additional file 8: Figure S2. — Gene networks visualized using Cytoscape3.3 and generated from both up- (red) and down- (blue) regulated gene sets in miR-27 KD neurons. (TIF 1178 kb) [file 12864_2016_3139_MOESM8_ESM.tif]

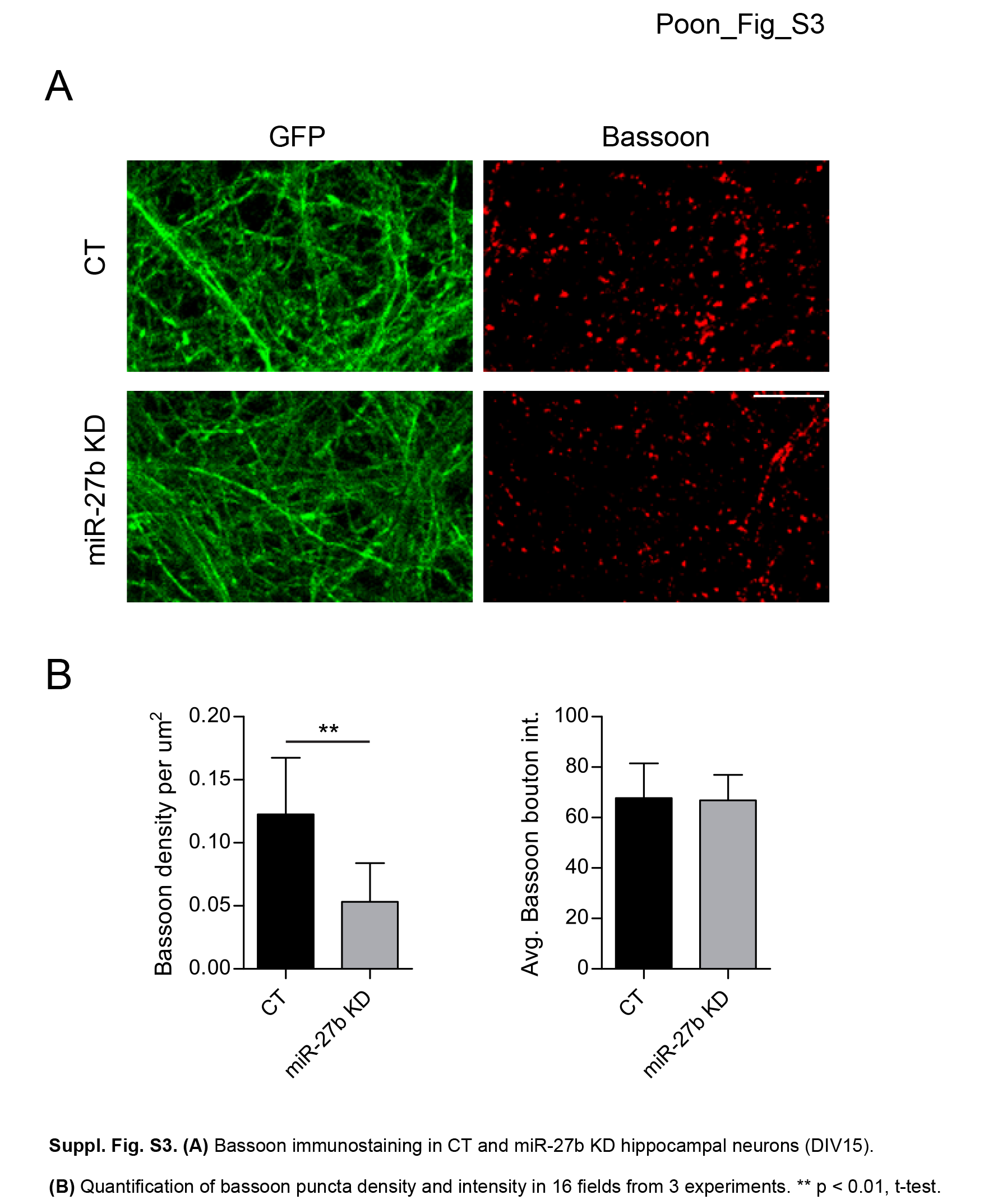

Supplement: Additional file 9: Figure S3. — Bassoon immunostaining in miR-27 KD and CT hippocampal neurons. (TIF 765 kb) [file 12864_2016_3139_MOESM9_ESM.tif]
